# Estimating the population-level prevalence of antimicrobial-resistant enteric bacteria from latrine samples

**DOI:** 10.1186/s13756-022-01145-4

**Published:** 2022-08-20

**Authors:** Sylvia Omulo, Maina Mugoh, Joshua Obiya, Moshe Alando, Douglas R. Call

**Affiliations:** 1 Paul G. Allen School for Global Health, Washington State University, Pullman, WA USA; 2 Washington State University Global Health-Kenya, Nairobi, Kenya; 3 University of Nairobi Institute of Tropical and Infectious Diseases, Nairobi, Kenya; 4 Kenya Medical Research Institute, Kisumu, Kenya; 5 Kenya Medical Research Institute, Nairobi, Kenya; 6 The Nelson Mandela African Institute of Science and Technology, Arusha, Tanzania

**Keywords:** Antimicrobial resistance, Latrines, Surveillance, Prevalence estimates

## Abstract

**Background:**

Logistical and economic barriers hamper community-level surveillance for antimicrobial-resistant bacteria in low-income countries. Latrines are commonly used in these settings and offer a low-cost source of surveillance samples. It is unclear, however, whether antimicrobial resistance prevalence estimates from latrine samples reflect estimates generated from randomly sampled people.

**Methods:**

We compared the prevalence of antimicrobial-resistant enteric bacteria from stool samples of people residing in randomly selected households within Kibera—an informal urban settlement in Kenya—to estimates from latrine samples within the same community. Fecal samples were collected between November 2015 and Jan 2016. Presumptive *Escherichia coli* isolates were collected from each household stool sample (n = 24) and each latrine sample (n = 48), resulting in 8935 and 8210 isolates, respectively. Isolates were tested for resistance to nine antibiotics using the replica-plating technique. Correlation- and Kolmogorov–Smirnov (K–S) tests were used to compare results.

**Results:**

Overall, the prevalence values obtained from latrine samples closely reflected those from stool samples, particularly for low-prevalence (< 15%) resistance phenotypes. Similarly, the distribution of resistance phenotypes was similar between latrine and household samples (*r* > 0.6; K–S *p*-values > 0.05).

**Conclusions:**

Although latrine samples did not perfectly estimate household antimicrobial resistance prevalence, they were highly correlated and thus could be employed as low-cost samples to monitor trends in antimicrobial resistance, detect the emergence of new resistance phenotypes and assess the impact of community interventions.

**Supplementary Information:**

The online version contains supplementary material available at 10.1186/s13756-022-01145-4.

## Background

Bacterial resistance to antibiotics, or antimicrobial resistance (AMR), remains one of the greatest global health challenges [1]. AMR surveillance enables us to track the emergence, prevalence, spread and persistence of resistant microorganisms. Nevertheless, apart from North America, Europe, and Canada, where surveillance—albeit passive—is routine, technical, logistical and/or financial barriers limit such efforts in low- and middle-income countries (LMICs) [2]. Additionally, when small-scale surveillance is conducted, it is unlikely that random sampling is employed, and informal settlements (slums) and rural populations may be missed entirely despite representing a potentially large proportion of the population. Consequently, less is known about the prevalence or long-term AMR trends in these communities.

Generating accurate and unbiased estimates of AMR at the community level requires that individuals (or households) be randomly selected for testing. Depending on how this is implemented, community AMR surveillance can be prohibitively expensive and out-of-reach for public health systems in LMICs, particularly where there are already significant challenges in securing sufficient resources for priorities such as basic hospital diagnostics. One way to mitigate this challenge is to identify low-cost, innovative alternatives for surveillance that can take advantage of existing resources.

**Fig. 1:**
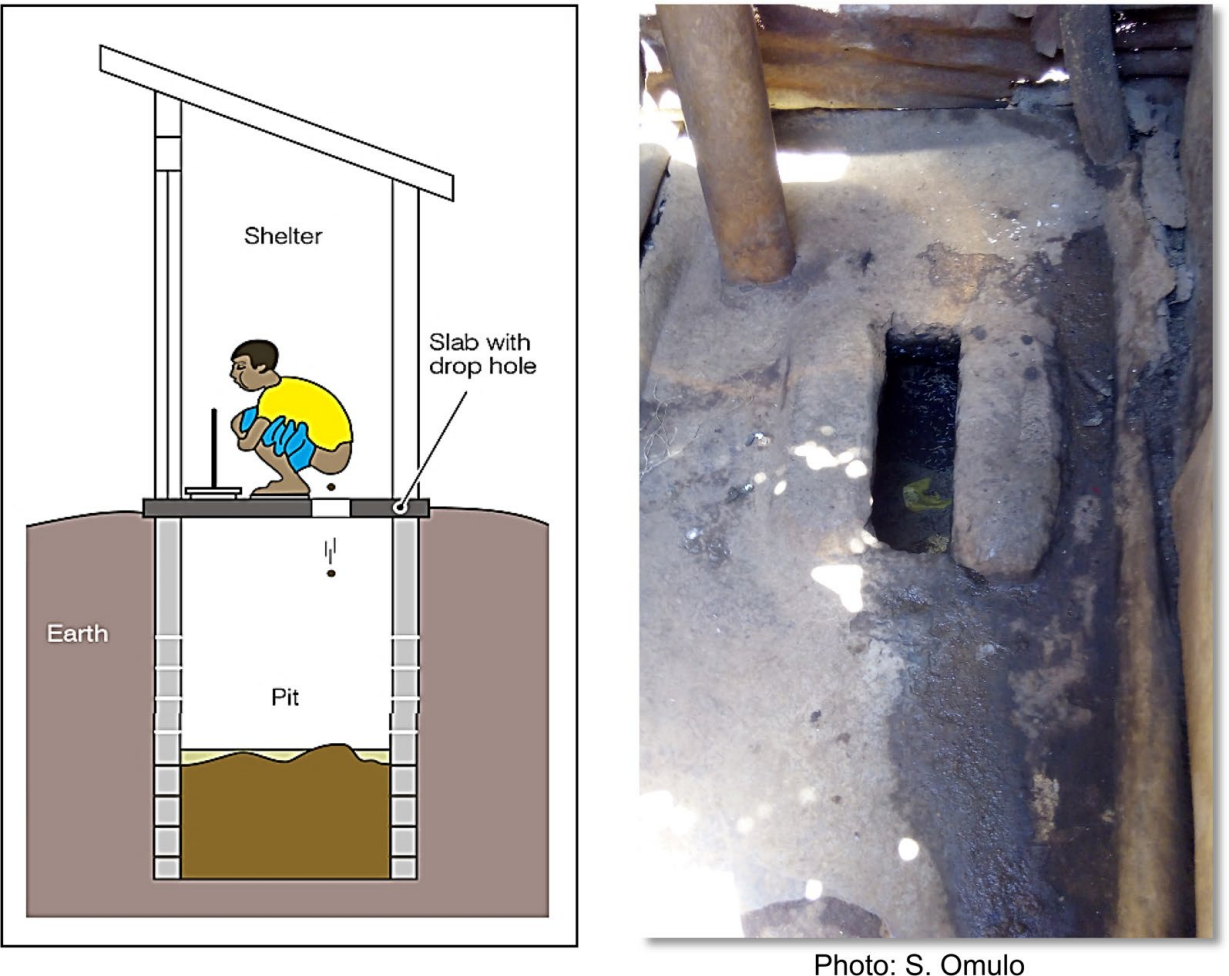
The general structure of a pit latrine, i.e., a hole dug into the ground for disposal of excreta (left) and the floor of a shallow concrete slab pit latrine in Kibera (right). Source: https://en.wikipedia.org/wiki/Pit_latrine

A common characteristic among informal settlements and rural populations is the lack of toilets within individual households. These populations rely on public pit latrines (Fig. 1) that are shared by multiple households [3]. Unsecured waste disposal systems can contaminate the environment—including soil or groundwater—and serve as foci for bacterial transmission between people, people and animals, and people, animals, and the environment [3–5]. Nevertheless, from a surveillance perspective, shared latrines offer pooled fecal samples that are easily collected and can routinely be tested using conventional microbiological methods, including low-cost and relatively high-throughput breakpoint assays.

The study aimed to determine if latrine samples can be used to estimate the prevalence of AMR in an informal urban settlement in Nairobi, Kenya. Latrine estimates were compared against a “gold standard”, i.e., stool samples collected from people residing in randomly selected households within the same community. We employed a breakpoint assay [5], which enabled us to characterize thousands of isolates at a limited cost without compromising detection sensitivity.

## Methods

This study was conducted within two densely-populated villages (Soweto: 55,000 persons/km, Gatwekera: 84,000 persons/km) [6] located in Kibera, an informal settlement in Nairobi. Latrines were sampled three times—once every two weeks—between November and mid-December 2015 and once in January 2016. This period was a wet season with 0.21–0.35 mm/day average rainfall [7] and corresponded with a period during which a longitudinal study involving biweekly visits to 200 randomly selected households was underway [3]. Shallow earthen or cemented latrines (Fig. 1) located within a 20-m radius of enrolled households were selected for sampling. These latrines were distributed throughout the 0.4 km^2^ study area. Samples were retrieved by dipping ~ 30 cm of a fresh 1.5 m long wooden stick into the center of each latrine’s pit, stirring the contents for 10 s and transferring adherent fecal material into a clean stool cap. To maintain consistency with the methods employed by the longitudinal study, samples were stored in chilled cooler boxes, transferred within 4 h of collection to a study lab located in Kibera, and processed on the same day.

The sample processing procedures used in this study have been described for the longitudinal study [3]. Briefly, a slurry containing 1 g of fecal sample and 9 mL of phosphate-buffered saline was prepared and serially diluted (tenfold). Glass beads were then used to spread 50 μL of the 10^–6^ dilution onto 90-mm MacConkey agar plates that had no antibiotics added. The plates were incubated at 37 °C overnight (18–24 h). To increase the probability of observing less common resistance phenotypes [8] while not biasing selection to some phenotypes, 48 presumptive *E. coli* isolates were collected from each agar plate used for latrine samples and 24 isolates from agar plates used for household stool samples. These sample sizes permitted > 90% probability of detecting rare resistance phenotypes at the sample level, assuming a true prevalence of 5% among latrine samples and 10% among household stool samples [8]. Collectively, over 17,000 isolates were characterized, providing a > 90% probability of detecting rare resistance phenotypes assuming a true prevalence of 0.02% [8].

Sterile toothpicks were used to transfer isolates from agar plates into individual wells of 96-well microtiter plates that contained 150 mL of Luria–Bertani (LB) media. After overnight incubation, a sterile pin replicator was used to transfer ~ 2 µL of culture from each well onto 150-mm MacConkey agar plates that included one of nine different antibiotics (ampicillin, Amp, 32 ug/mL; ceftazidime, Caz, 8 ug/mL; chloramphenicol, Chl, 32 ug/mL; ciprofloxacin, Cip, 4 ug/mL; kanamycin, Kan, 64 ug/mL; streptomycin, Str, 16 ug/mL; sulfamethoxazole, Sul, 512 ug/mL; tetracycline, Tet, 16 ug/mL; and trimethoprim, Tmp, 16 ug/mL, all from Sigma, St. Louis, MO). Complete growth of a colony indicated resistance to the respective antibiotic, otherwise, susceptibility. Antibiotic concentrations were based on Clinical and Laboratory Standards Institute minimum inhibitory concentrations standards for Enterobacteriaceae [9]. A detailed analysis of the diagnostic sensitivity and specificity of the breakpoint assay (used in this study) relative to whole-genome sequencing (correspondence between phenotype and genotype) is provided by Subbiah et al*.* [5].

Fifty latrines were targeted for sampling, but we limited correlation analyses to latrines sampled on at least three visits (a single sample was collected per visit). The proportion of resistant isolates from each sample and the resistance profiles (antibiotic combinations to which an isolate was resistant) were then determined as described above. Based on the observed data, we considered resistance values < 15% as “low prevalence” and those ≥ 15% as “high prevalence”. The correlation between the prevalence estimates for latrine and household stool samples and the proportional distribution of resistance profiles were analyzed using Fisher’s correlation- and Kolmogorov–Smirnov (K–S) tests, respectively. Sample sources were considered highly comparable at correlation values > 0.8 and K–S *p*-values > 0.05, or if the 95% confidence intervals (CIs) overlapped.

## Results

Forty-six (out of 50) latrines and 121 (out of 174) households were sampled at least three times between Nov 2015 and Jan 2016. Cumulatively, 8210 presumptive *E. coli* isolates were collected from latrines and 8935 isolates from household stool samples during the four sampling rounds (Table 1).

**Table 1:** Sources of samples (latrine or household (HH) stool) and the aggregate number of presumptive *E. coli* isolated from each sample source during the four rounds* of sampling

	Latrines sampled	*E. coli* isolated	Households sampled	*E. coli* isolated
Round 1	41	1946	115	2456
Round 2	46	2184	116	2429
Round 3	43	2042	100	2057
Round 4	43	2038	95	1993
Total		8210		8935

^*^Sampling dates: Round 1: (9–20 Nov 2015); Round 2: (23 Nov-4 Dec 2015); Round 3: (7–18 Dec 2015); Round 4: (11–22 Jan 2016)

**Fig. 2:**
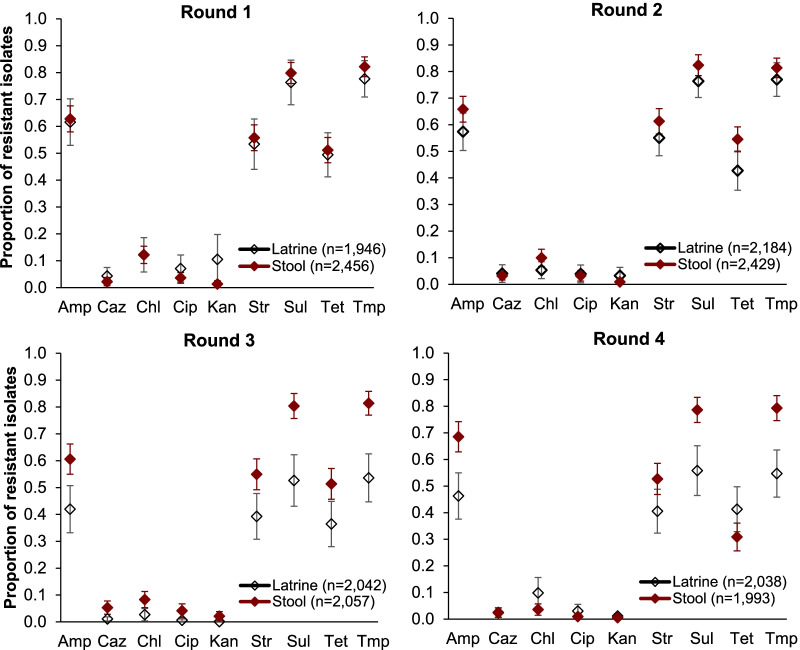
Prevalence estimates (mean and 95% CI) from latrine and stool samples over four rounds of sampling conducted two weeks apart (between rounds 1–2 and 2–3) and four weeks apart (rounds 3–4). Ampicillin (Amp), ceftazidime (Caz), chloramphenicol (Chl), ciprofloxacin (Cip), kanamycin (Kan), streptomycin (Str), sulfamethoxazole (Sul), tetracycline (Tet), trimethoprim (Tmp). Red diamonds indicate stool samples, while white diamonds indicate latrine samples

The prevalence of antimicrobial-resistant *E. coli* isolated from latrines used by households living within Kibera was highly correlated with the prevalence of antimicrobial-resistant *E. coli* in household stool samples (*r*^2^ = 0.99; *p* < 0.001). Nevertheless, 5–30% differences were observed in individual sampling rounds for antibiotics whose prevalence values were > 20% (ampicillin, streptomycin, sulfamethoxazole, tetracycline, and trimethoprim). For ceftazidime, chloramphenicol, ciprofloxacin, and kanamycin, whose resistance prevalence ranged between one and 15%, estimates from latrine samples very closely mirrored those from household stool samples (Fig. 2).

**Fig. 3:**
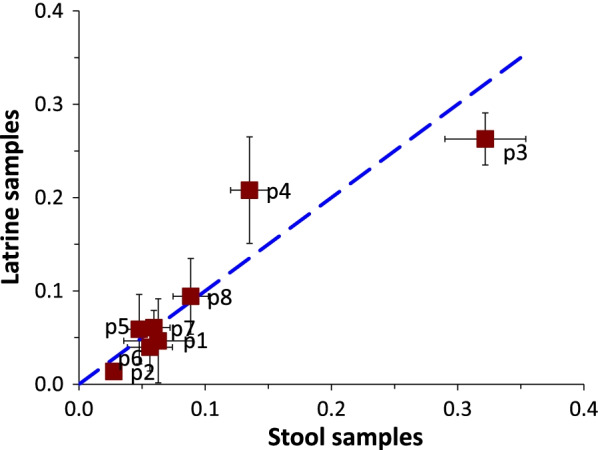
Aggregate proportions (mean and 95% CI) of the eight most abundant resistance profiles identified in latrine and stool sample isolates over the sampling period. The dotted line represents the proportion at which prevalence values in latrine and stool samples are equal. Points above this line have greater representation in latrine samples while points below this line have greater representation in stool samples

The proportional distribution of latrine samples with resistance to one, two, three or more (n = 9) antibiotic combinations mirrored that of household stool samples, although stool samples had more isolates that were resistant to three, four and five antibiotics. There were more susceptible isolates in latrine than stool samples (29% vs. 13%, respectively; see Additional file 1). In total, 136 multidrug resistance phenotypes were detected in latrine samples and 141 in household stool samples. Of these, 21 phenotypes (see Additional file 2) were detected in all sampling rounds and both sources, eight of which had the highest abundance (Table 2, left pane). When analyzed for correlation and proportional distribution by the K–S test, all 21 phenotypes showed a strong correlation in most sampling rounds (Table 2, right pane). The proportional distributions of the eight most abundant phenotypes (p1–p8) in latrine and household stool samples were highly similar except for p3, which was marginally higher in stool samples, and p4 in latrine samples, which was statistically different as the 95% CIs did not overlap (Fig. 3).

**Table 2:** Left pane: abundant resistance phenotypes (p1-p8); Right pane: Correlation and K–S p-values for all [21] phenotypes identified in latrine and stool samples

Label	Phenotype	Correlation (n = 21)
p1	AmpChlStrSulTetTmp	Round 1 (0.97)
p2	AmpChlStrSulTmp	Round 2 (0.91)
p3	AmpStrSulTetTmp	Round 3 (0.89)
p4	AmpStrSulTmp	Round 4 (0.60)
p5	AmpSulTetTmp	**K–S test *p*-value (n = 21)**
p6	AmpSulTmp	Round 1 (0.84)
p7	SulTetTmp	Round 2 (0.98)
p8	SulTmp	Round 3 (0.84)
		Round 4 (0.09)

A correlation value of one (1) represents perfect correlation; K–S *P*-values > 0.05 indicate samples have similar statistical distributions

*Amp* Ampicillin, *Chl* chloramphenicol, *Str* streptomycin, *Sul* sulfamethoxazole, *Tet* tetracycline, *Tmp* trimethoprim

## Discussion

Various studies have exploited sewage [10] and/or toilet sampling [11–14] to demonstrate the presence of antimicrobial-resistant organisms and highlight these sources’ potential to transmit disease and antimicrobial resistance genes. However, few (if any) have directly compared AMR estimates obtained from these sources with presumptively unbiased, randomly selected stool samples from the same community. For example, the Global Sewage Project [10] characterizes sewage from around the world, providing one-time estimates of the prevalence of AMR based on molecular detection of antibiotic-resistance genes. However, it does this without assessing potential biases relative to randomly sampled households. Similarly, a recent study conducted in South Africa quantified the presence and prevalence of *E. coli* [12] and *Staphylococcus aureus* [14] in pit latrines and compared these with other published data collected from clinical isolates within the same country but did not consider comparisons with an unbiased sampling design. Our study capitalized on an ongoing longitudinal study to determine how closely latrine samples estimate the community prevalence of AMR compared to household stool samples. By concurrently and repeatedly sampling latrines during periods when longitudinal household sampling occurred, we were able to assess how AMR prevalence estimates varied over a span of three months.

Latrine samples provided similar estimates to stool samples for the prevalence of AMR at the community level and for the types and proportional distributions of circulating AMR phenotypes. These estimates were markedly similar when resistance prevalence estimates were < 15% (ceftazidime, chloramphenicol, ciprofloxacin, and kanamycin), but varied for higher prevalence types (ampicillin, streptomycin, sulfamethoxazole, tetracycline, and trimethoprim). The rainfall experienced during the sampling months may have contributed to the dilution of latrine samples. Further, there may have been differential survival of different resistant strains. For example, beta-lactamase genes are typically expressed constitutively [15], whereas tetracycline resistance genes are typically regulated by a repressor protein and will only express in the presence of tetracycline antibiotics [16]. The variations observed between sampling Rounds 3 and 4 may have resulted from changes in latrine use and household composition due to the influx and outflux of individuals visiting relatives during holiday festivities [3]. Nevertheless, the finding that latrine samples very closely estimated low-prevalence resistance indicates that these samples can reliably be used for surveillance, particularly for bacteria with emerging resistance phenotypes, which commonly occur at low levels when first introduced. For example, ciprofloxacin is rarely used in Kibera, while ceftriaxone and chloramphenicol use is unreported [3], consistent with the observed phenotypes. Changes in these phenotypes could indicate shifts in community antibiotic use, increased hospital-to-community transmission of resistant bacteria, or environmental factors that could favor emergent phenotypes. Estimating highly prevalent resistance types may require broader and repeated sampling for a more robust estimate of the true community-level prevalence of antimicrobial-resistant bacteria in stool. Surveillance may prioritize resistance phenotypes that are most impacted by community antibiotic use or those indicated in AMR national action plans.

AMR surveillance remains a challenge in LMICs where economic resources are constrained; surveillance investments compete directly with the delivery of healthcare services. Our study demonstrates that latrine samples can be useful for routine surveillance of circulating AMR strains, particularly among rural populations, informal settlement communities, and migrant populations, while requiring relatively minimal infrastructural investment, reagent needs and specialist training. With approximately 3.4 billion people living in rural areas [17], and 0.9 billion in informal settlements [18], > 70% of whom use latrines or practice open defecation [19], latrine and other environmental samples may not only be readily available but can also provide accurate population-level AMR prevalence estimates for a large proportion of the global population.

One application of such surveillance is monitoring trends over time. For example, within a given geographic region, systematic surveillance of latrines could provide robust prevalence estimates needed to understand how resistance levels change in response to interventions and policy changes that arise from national antimicrobial resistance action plans, education campaigns, or altered access to healthcare services. Latrine surveillance could also be used to alert the medical community when new resistance phenotypes are emerging in a population and identify how antimicrobial-resistant bacteria are introduced into the environment or different host populations. The temporal order in which emerging resistance appears would help determine the origin of the resistance traits (e.g., the transmission of resistant bacteria from human to food animal or vice versa), which is very difficult to do once these traits are found in both human and animal populations.

We acknowledge certain limitations of this study. Firstly, by limiting our focus on *E. coli* to allow us to compare data from latrine samples with household stool samples (colonizing *E. coli* strains), we cannot generalize the applicability of this surveillance approach to pathogenic *E. coli* or other enteric bacteria that can also survive in raw sewage [20–22]. Nevertheless, *E. coli* remains a convenient choice for AMR surveillance in community settings. Secondly, by using a moderately high-throughput testing methodology (i.e., breakpoint assays), less prevalent but potentially medically important resistance phenotypes (e.g., carbapenem resistance) may be missed simply by chance. This shortcoming could be overcome by impregnating agar plates with desired antibiotics. The choice of antibiotics used in this study partially reflects resistance phenotypes that can be impacted by antibiotic use in Kibera [3], and panels used in other studies [5, 23–25]. Antibiotic selection can be tailored to suit any given surveillance program so long as the selected antibiotics are compatible with agar-based assays (e.g., colistin cannot be used in this format). Thirdly, although the diagnostic sensitivity of our panel of nine antibiotics was > 0.91, the diagnostic specificity (true positives) varied by antibiotic (up to 0.93 for tetracycline and trimethoprim). The most common resistance phenotype for which specificity was low (streptomycin, 0.75), likely due to insufficient antibiotic concentration in the media. Despite this variation, our team has had sufficient precision using the replica plating method to partition the variance amongst risk factors for colonization with antibiotic-resistant bacteria [5, 23]. Lastly, by analyzing presumptive *E. coli*, we likely faced some level of identification error. We surmise that this error was minimal based on other analyses conducted on these isolates [24]. This approach offers the advantage of maintaining costs low while allowing the collection and characterization of many isolates.

## Conclusion

Latrine samples closely approximate household stool samples in prevalence and phenotype distribution. Although they do not perfectly reflect the gold standard, they provide a means to easily monitor trends over time with a high degree of confidence. Differences between latrine and stool averages in the last two sampling rounds imply that single sampling campaigns may yield biased estimates and that multiple sampling rounds may generate more accurate AMR estimates for a population. Over time, latrine samples should be suitable for identifying shifts in prevalence and detecting the emergence of novel resistance phenotypes at a much lower cost than household-level surveys.

## Supplementary Information

**Additional file 1**. Aggregate distribution of antimicrobial resistance profiles identified in latrine and stool samples.

**Additional file 2**. The 21 phenotypes that were detected in all four sampling rounds (R1-4) and in both sources (i.e., latrine and stool samples).

## Data Availability

The datasets used and/or analyzed during the current study are available from the corresponding author on reasonable request.

## Abbreviations

AMR: Antimicrobial resistance
CDC: Centers for Disease Control and Prevention
CI: Confidence interval
HH: Household
IRB: Institutional Review Board
KEMRI: Kenya Medical Research Institute
K–S: Kolmogorov–Smirnov
LB: Luria Bertani
LMICs: Low- and middle-income countries
SERU: Scientific and Ethics Review Unit
