# Supplementary material for: Estimating the population-level prevalence of antimicrobial-resistant enteric bacteria from latrine samples
Source: Antimicrob Resist Infect Control. 2022 Aug 20;11:106. doi: 10.1186/s13756-022-01145-4 (PMC9392229; doi:10.1186/s13756-022-01145-4)
Supplement: Supplementary file 1 — Additional file 1. Aggregate distribution of antimicrobial resistance profiles identified in latrine and stool samples. [file 13756_2022_1145_MOESM1_ESM.docx]

**Additional file 1:** Aggregate distribution of antimicrobial resistance profiles identified in latrine and stool samples.
