# Supplementary material for: Estimating the population-level prevalence of antimicrobial-resistant enteric bacteria from latrine samples
Source: Antimicrob Resist Infect Control. 2022 Aug 20;11:106. doi: 10.1186/s13756-022-01145-4 (PMC9392229; doi:10.1186/s13756-022-01145-4)
Supplement: Supplementary file 2 — Additional file 2. The 21 phenotypes that were detected in all four sampling rounds (R1-4) and in both sources (i.e., latrine and stool samples). [file 13756_2022_1145_MOESM2_ESM.docx]

**Additional file 2**: The 21 phenotypes that were detected in all four sampling rounds (R1-4) and in both sources (i.e., latrine and stool samples). Numbers indicate the number of isolates with the corresponding phenotype.

|  | **Latrine samples** | | | |  | **Stool samples** | | | |
| --- | --- | --- | --- | --- | --- | --- | --- | --- | --- |
|  | **R1**  (N = 1,070) | **R2**  (N = 1,687) | **R3**  (N = 1,201) | **R4**  (N = 1,019) |  | **R1**  (N = 2,521) | **R2**  (N = 2,268) | **R3**  (N = 1,665) | **R4**  (N = 1,893) |
|  | **n (%)** | **n (%)** | **n (%)** | **n (%)** |  | **n (%)** | **n (%)** | **n (%)** | **n (%)** |
| Amp | 11 (1) | 16 (1) | 14 (1) | 17 (2) |  | 21 (1) | 27 (1) | 9 (1) | 142 (7) |
| AmpCazCipStrSulTetTmp | 1 (0) | 1 (0) | 2 (0) | 2 (0) |  | 20 (1) | 14 (1) | 14 (1) | 1 (0) |
| AmpCazStrSulTetTmp | 2 (0) | 4 (0) | 18 (1) | 17 (2) |  | 9 (0) | 3 (0) | 17 (1) | 2 (0) |
| AmpChlStrSulTetTmp | 104 (9) | 59 (3) | 20 (2) | 73 (7) |  | 224 (9) | 150 (6) | 68 (4) | 54 (3) |
| AmpChlStrSulTmp | 22 (2) | 28 (2) | 8 (1) | 22 (2) |  | 73 (3) | 52 (2) | 57 (3) | 2 (0) |
| AmpChlSulTetTmp | 3 (0) | 2 (0) | 1 (0) | 25 (2) |  | 2 (0) | 19 (1) | 14 (1) | 8 (0) |
| AmpCipStrSulTetTmp | 3 (0) | 6 (0) | 4 (0) | 8 (1) |  | 4 (0) | 32 (1) | 18 (1) | 11 (1) |
| AmpStrSulTet | 6 (1) | 21 (1) | 34 (3) | 16 (1) |  | 10 (0) | 15 (1) | 11 (1) | 13 (1) |
| AmpStrSulTetTmp | 327 (28) | 472 (27) | 300 (24) | 323 (30) |  | 843 (32) | 828 (35) | 510 (29) | 277 (15) |
| AmpStrSulTmp | 174 (15) | 408 (23) | 306 (24) | 125 (12) |  | 316 (12) | 351 (15) | 237 (14) | 608 (32) |
| AmpSulTetTmp | 67 (6) | 48 (3) | 116 (9) | 95 (9) |  | 101 (4) | 114 (5) | 98 (6) | 178 (9) |
| AmpSulTmp | 68 (6) | 78 (4) | 20 (2) | 20 (2) |  | 186 (7) | 97 (4) | 98 (6) | 137 (7) |
| AmpTetTmp | 3 (0) | 2 (0) | 4 (0) | 16 (1) |  | 13 (1) | 21 (1) | 12 (1) | 12 (1) |
| StrSul | 3 (0) | 2 (0) | 1 (0) | 4 (0) |  | 2 (0) | 1 (0) | 2 (0) | 1 (0) |
| StrSulTet | 1 (0) | 14 (1) | 9 (1) | 3 (0) |  | 3 (0) | 6 (0) | 12 (1) | 2 (0) |
| StrSulTetTmp | 21 (2) | 72 (4) | 102 (8) | 37 (3) |  | 99 (4) | 118 (5) | 123 (7) | 31 (2) |
| StrSulTmp | 24 (2) | 21 (1) | 8 (1) | 15 (1) |  | 63 (2) | 80 (3) | 54 (3) | 151 (8) |
| SulTetTmp | 89 (8) | 104 (6) | 58 (5) | 92 (9) |  | 145 (6) | 120 (5) | 125 (7) | 73 (4) |
| SulTmp | 79 (7) | 237 (13) | 101 (8) | 83 (8) |  | 259 (10) | 180 (8) | 157 (9) | 176 (9) |
| Tet | 5 (0) | 25 (1) | 32 (3) | 10 (1) |  | 56 (2) | 20 (1) | 11 (1) | 8 (0) |
| Tmp | 57 (5) | 67 (4) | 43 (3) | 16 (1) |  | 72 (3) | 20 (1) | 18 (1) | 6 (0) |
